# Stress-Induced “Immediate” Lactate (iBLC) Response Differences in Pubertal and Young Adult Soccer Players

**DOI:** 10.3390/sports13110384

**Published:** 2025-11-04

**Authors:** Ferenc Ihász, Ottó Vincze, Imre Soós, István Barthalos, Zoltán Alföldi, Anna Horváth Pápai, Ádám Balog, László Suszter

**Affiliations:** 1 Department of Health Promotion and Exercise Science, Széchenyi István University, H-9026 Győr, Hungary; ihasz.ferenc@sze.hu (F.I.); barthalos.istvan@sze.hu (I.B.); alfoldi.zoltan@sze.hu (Z.A.); 2 Doctoral School of Health Sciences, University of Pécs, H-7624 Pécs, Hungary; vincze@eto.hu (O.V.); imre.soos@etk.pte.hu (I.S.); papai.anna@gmail.com (A.H.P.); balogadam85@gmail.com (Á.B.); 3 Sport and Health Sciences Research Group, Eszterházy Károly Catholic University, H-3300 Eger, Hungary

**Keywords:** blood lactate response, youth soccer players, position-specific performance

## Abstract

**Background**: High-intensity physical activity elicits acute physiological responses across the metabolic, cardiovascular, respiratory, endocrine, immune, and neuromuscular systems. During recovery, multiple processes act to restore homeostasis and functional capacity. The aim of this study was to examine age- and position-related differences in cardiovascular and metabolic responses among youth soccer players. **Methods**: A total of 147 male academy soccer players were assessed, divided into four age groups (U15, U16, U17, U18/U19) and four playing positions (defenders, midfielders, forwards, goalkeepers). **Results**: Significant anthropometric and physiological differences were observed between age groups. Body height and weight increased progressively with age (*p* < 0.05), while body fat percentage was lower in younger compared to older cohorts (*p* < 0.001). Relative muscle mass did not differ significantly between groups. Absolute aerobic capacity (VO_2_max) was higher in U17–U19 compared to U15–U16 (*p* < 0.001). Heart rate at anaerobic threshold (HRAT) and maximal heart rate were greater in the younger groups (*p* ≤ 0.005). Immediate blood lactate (iBLC) and ΔiBLC were significantly higher in U15–U16 compared to U17–U19 (both *p* < 0.001). Position-specific analysis revealed higher iBLC in U15 defenders compared to U18/U19 defenders (*p* < 0.01), whereas no positional differences were observed in relative VO_2_max. **Conclusions**: These results are due to a carefully designed training program and frequent individual training sessions.

## 1. Introduction

During short-duration (2–9 min) high-intensity physical activity, children rely more heavily on oxidative metabolism than adults [1]. Prepubertal children (<12 years) typically present lower resting skeletal muscle glycogen stores than adults, with values increasing through adolescence. In addition, previous studies have demonstrated age-related differences in intramuscular phosphocreatine kinetics, with children showing smaller depletion and faster resynthesis, which may attenuate the glycolytic contribution during intense exercise [2,3,4]. Consequently, these reserves are mobilized to a lesser extent, which may result in lower lactate production. It is essential to emphasize the importance of acid–base balance [5], which aligns with children’s lower glycolytic capacity and the lower peak blood lactate (BL) values observed after exercise [6,7]. It is nevertheless conceivable that muscle metabolism differs substantially between children and adults [8]. Some researchers attribute age-related differences in performance to metabolic factors [8], while others emphasize limited neuromotor unit mobilization [9].

The acute physiological responses of adolescents and young adults to intense exercise differ from those of adults across multiple domains, including the cardiovascular, respiratory, and metabolic systems. During exercise, children and adolescents rely to a greater extent on oxidative pathways, whereas their glycolytic capacity and intramuscular glycogen–phosphocreatine stores are relatively more limited. As a consequence, they typically show lower peak blood lactate and faster recovery [2,10]. These differences can be explained in part by maturation-related neuromuscular factors—notably, motor unit recruitment and muscle fiber-type composition [11]. During puberty, hormonal changes—especially increases in testosterone and growth hormone—exert substantial effects on muscle mass accretion and oxygen transport capacity [12]. In parallel, increases in red blood cell count and hemoglobin concentration support gains in VO_2_max; however, structural adaptations in muscle, changes in body composition, and hormonal influences also contribute significantly to aerobic capacity development during growth [13,14].

Research on lactate response across different age groups consistently indicates that prepubertal children (<12 years) accumulate less lactate in their blood during exercise and may exhibit faster lactate clearance than adolescents (13–17 years) or adults (>18 years) [4,15]. A substantial portion of these discrepancies may not stem from differences in the glycolytic capacity of skeletal muscle per se, but rather from the dynamics of blood–tissue transit time and removal processes [15]. In boys (prepubertal and early pubertal, typically <15 years), the peak of blood lactate concentration (BLC) often occurs earlier than in adult men (>18 years), suggesting a shift in the phase of BLC kinetics [16,17].

The sport specific-load profile of football (soccer), in which both aerobic and anaerobic energy systems are critical, further nuances this picture. Among outfield players, midfielders typically cover greater total distances and display relatively higher endurance indicators, whereas goalkeepers show the lowest endurance capacity [18,19]. However, the relationship between VO_2_max and in-match running performance is not invariably direct, highlighting the roles of position, tactics, and context [20]. Notably, the international literature has focused primarily on adult professional footballers [21,22], leaving limited data on position-specific metabolic profiles in youth players.

These considerations motivate a comparative analysis of stress-induced “immediate” blood lactate (iBLC) and related cardiometabolic indicators by age group and playing position in adolescent and young adult footballers. Accordingly, the present study aimed to examine age- and position-related differences (defender, midfielder, forward, goalkeeper) in aerobic capacity (VO_2_max), heart rate at the anaerobic threshold (HRAT), resting and post-exercise blood lactate (rBLC, iBLC), and the dynamics of the iBLC response (ΔiBLC), while accounting for age-specific lactate response and the position-specific loading patterns of football. We hypothesized that the faster lactate response and more favorable oxidative profile in younger cohorts would result in distinct position-specific iBLC patterns, with practical implications for training prescription and recovery management.

## 2. Materials and Methods

The study involved 147 boys who were soccer players in the education system. We examined four age groups—U15, *n* = 49; U16, *n* = 45; U17, *n* = 30; U18-U19, *n* = 23—and four positions—defenders (*n* = 49), midfielders (*n* = 40), forwards (*n* = 41), and goalkeepers (*n* = 20) (Table 1). Each participant was included only once and assigned exclusively to the age group corresponding to his chronological age. A trained anthropometrist took all the anthropometric measurements in accordance with the standardized procedures of the International Society for the Advancement of Kinanthropometry (ISAK, Level 1) [23]. We measured body height (BH) and body weight (BW), as well as body composition: body fat mass (F%) and skeletal muscle mass (M%) using the seca medical Body Composition Analyzer (mBCA 515, seca GmbH & Co.). The device operates on the principle of bioelectrical impedance analysis (BIA). This foot-to-foot, hand-to-hand, and hand-to-foot contact device has two stainless steel footrest electrodes and two handles, enabling tetrapolar eight-point contact. The reliability of bioelectrical impedance analysis has been successfully demonstrated in comparison with other body composition measurement methods, such as DXA [24].

The stress tests were conducted in the stress physiology laboratory of the Fehér Miklós Football Academy using Piston Ltd. European VAT code: HU 10,465,905 equipment during the fall season. The test was performed on a treadmill based on a progressive intensity protocol until complete exhaustion. The ergospiroemetric tests were performed before the start of the competition season, following a progressive intensity protocol until voluntary exhaustion (failure) on a motorized treadmill (Pulsar 4.0, h/p/Cosmos Sports & Medical GmbH, Nussdorf-Traunstein, Germany). Before starting the exercise, the players performed an individual warm-up consisting of 3 min of cycling at their own pace and 2 min of dynamic stretching. The test protocol began with walking at 5 km/h for one minute, then continued at a speed of 8 km/h. The speed increased by 2 km/h every two minutes, with a continuous incline of 2°. a protocol supported by previous validation work indicating that such a moderate incline induces a meaningful but not excessive cardiometabolic load in athletes [25]. During the task, we recorded the resting heart rate (RHR) and maximum heart rate (HR_max_), using a chest transmitter and receiver (Garmin HRM3-SS Garmin Ltd., Olathe, KS., USA).

The following cardiorespiratory variables were monitored: heart rate (HR bpm), oxygen uptake (V̇O_2_ measured in mL/kg/min), carbon dioxide uptake (V̇CO_2_ L/min), respiratory exchange ratio (RER, arbitrary units; AU) expressed as the ratio of the two metabolites (V̇CO_2_/V̇O_2_), and relative oxygen uptake (rV̇O_2_) at the anaerobic threshold (AT) (rV̇O_2_/AT mL/kg/min).

The heart rate at anaerobic threshold (HRAT) was determined after completion of the exercise test; HRAT was determined for each subject using the V-slope method developed by Beaver et al. (1986) [26]. This method involves the analysis of the response of VCO_2_ to VO_2_ and assumes that the threshold value corresponds to the breakpoint of the VCO_2_/VO_2_ relationship and the corresponding HR, time spent on the treadmill (sec), maximum running speed (speed), and relative power output (rPO W/kg). The V̇O_2max_ value was accepted if at least 3 criteria were met: (1) HR in the last minute exceeded 95% of the subject’s age-predicted HRmax, which has been previously calculated according to Tanaka et al. (2001) [27]; (2) V̇O_2max_ was plateaued despite increasing treadmill speed, V̇O_2_ < 150 mL O_2_ [28]; (3) RER (V̇CO_2_/V̇O_2_) reached or exceeded 1.1 AU [29], and subjects were unable to continue running despite verbal encouragement.

Blood samples were taken from the earlobe before the start of the exercise and 2.5 min after its completion, Accutrend^®^ Plus, RR0068242, Roche, Mannheim, Germany. In line with several validated protocols in exercise physiology, particularly in youth populations, we applied a 2.5 min post-exercise sampling point, which enables direct comparison with previous studies while capturing the immediate metabolic response.

Data collection was fully compliant with the ethical principles of the Helsinki Declaration. Participants and their legal guardians were fully informed about the study, and written consent was obtained for participation. The study was conducted voluntarily in collaboration with the sports clubs and national rowing associations involved. All necessary ethical and procedural requirements were met. The study was approved by the Scientific Ethics Committee of the Scientific Advisory Board of Széchenyi István University (SZE/ETT-51/2025 (VIII.25), Hungary).

### Statistical Analyses

During descriptive analyses, mean ± standard deviation and relative frequencies were reported. To compare age groups (U15, U16, U17, U18/U19) and positions (defenders, midfielders, forwards, goalkeepers) on the variables measured anthropometric, body composition characteristics and laboratory tests one-way ANOVA was used with calculation of omega-squared effect size measurement for overall tests. Due to unequal sample sizes, F-value of the robust Welch’s ANOVA was reported. For multiple comparisons, Games-Howell test was used. The level of significance was set at 0.05, and for multiple testing, an adjusted *p*-value was used for the conclusion using Bonferroni correction. Statistical analyses were conducted using IBM SPSS Statistics for Windows, Version 27.0 (IBM Corp. Released 2020, Armonk, NY, USA). A priori power analysis was conducted to calculate the required sample size using G*Power 3.1.9.7. For F tests, ANOVA fixed effects, omnibus, one-way, the required sample size is 112 with the following parameters: f = 0.40 (large), α = 0.05, power = 0.95, number of groups = 4. The required sample size was respected in the present study.

## 3. Results

We included 147 soccer players in the study. For the sample size and relative frequencies by age groups and positions, see Table 1.

Anthropometric and body compositions characteristics revealed significant differences between age groups (Table 2). The U15 and U16 groups showed significantly lower body height and relative fat mass compared to the U18/U19 group. U17 group has an intermediate value (body height: F(3,135) = 5.785, *p* = 0.001, ω^2^ = 0.06; relative fat mass: F(3,135) = 8.272, *p* < 0.001, ω^2^ = 0.05). The U15 and U16 groups have a lower body weight compared to the U17 and U18/U19 groups (F(3,135) = 2.898, *p* = 0.042, ω^2^ = 0.12). Relative muscle mass did not show a significant difference between the age groups (F(3,135) = 0.572, *p* = 0.635, ω^2^ = 0.01).

Exercise tests revealed significant differences between the age groups. The U15 and U16 groups showed a lower absolute aerobic capacity (VO_2max_) and resting blood lactate (rBLC) and showed a higher heart rate at anaerobic threshold (HRAT), immediate blood lactate (IBLC), delta lactate (ΔIBLC), and maximal heart rate (MP) compared to U18/U19 and U17 (intermediate values, U17: rBLC, IBLC, MP; U18/U19: HRAT). There were non-significant differences between age groups in rVO_2max_ and RHR (Table 3).

**Figure 1:**
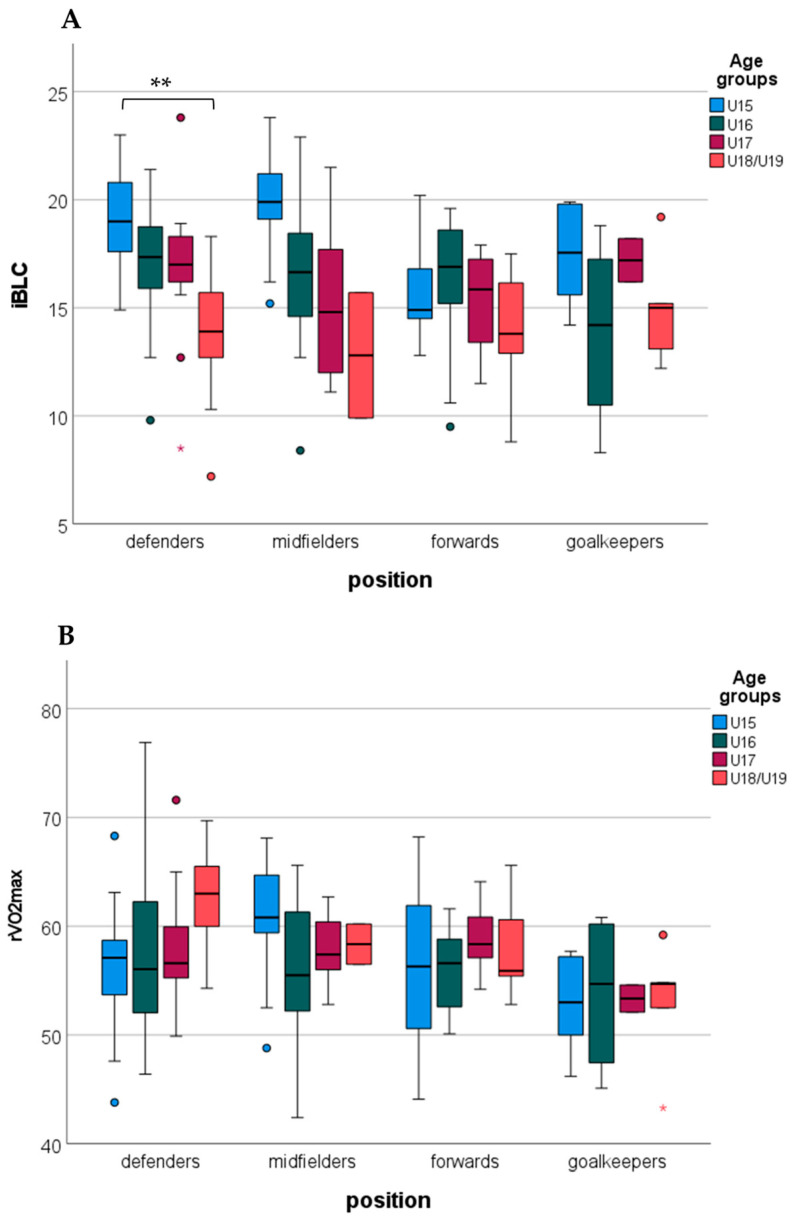
Differences in immediate blood lactate (iBLC, (**A**)) and relative aerobic capacity (rVO_2max_, (**B**)) between age groups by positions (**: *p* < 0.01; circles indicate outliers).

Examining the differences between age groups by the positions, immediate blood lactate revealed a significant difference between the age groups in defenders. The U15 group showed a higher immediate blood lactate compared to the U18/U19 groups. In other positions, immediate blood lactate showed a non-significant age group difference (Figure 1A). rVO_2max_ showed non-significant differences between the age groups in the different positions (Figure 1B).

## 4. Discussion

Our first important finding was that there was no significant difference in relative muscle mass between age groups, which contradicts previous reports of an age-related increase in serum lactate levels [10]. In this study, we analyzed male soccer players aged 15–19 years (N = 147) who trained twice daily within an academy system under a scientifically structured program and competed in league matches across both the fall and spring seasons. Our first key finding was that no significant differences were observed in relative muscle mass across the age groups. In contrast, significant differences in mean serum lactate were found among defenders, specifically between the youngest and the oldest groups. At the whole-sample level, mean serum lactate concentrations were inversely proportional to age. This pattern contradicts previous reports suggesting that serum lactate levels increase with age [10]. In the present cohort, the opposite trend was observed. No differences were detected between age groups in relative aerobic capacity (rVO_2_max), treadmill running time (Time), maximal running speed (Speedmax), or relative power output (rWatt). Accordingly, only limited evidence supports the notion that post-exercise blood lactate concentration (BLC) differences between adolescents and young adults reflect age- or maturation-related differences in skeletal muscle glycolytic capacity. In line with this interpretation, the highest BLC values have been reported when blood samples were obtained at 1 min intervals for up to 7 min post-exercise [30], suggesting that intergroup differences are more likely attributable to variations in lactate response rather than in intrinsic muscle metabolic profiles.

According to the literature, adolescents do not possess a “secret weapon” with respect to lactate metabolism [11]; rather, their responses appear to stem from distinct kinetic characteristics. Engel et al. [16] demonstrated that boys, on average, reached peak BLC approximately 2.6 min earlier than adult men, likely due in part to shorter circulation times and hemodynamic differences, although multiple mechanisms may contribute. Several authors have also suggested that adolescents may present with relatively greater proportions of type I fibers, which could reduce the recruitment of type II motor units [6]. Moreover, some of the observed age-related differences appear to be attributable not to glycolytic capacity per se, but rather to differences in blood–tissue transport times and clearance processes [15].

Consistent with these findings, we observed no significant age-related differences in key cardiovascular or metabolic parameters in our cohort, suggesting that a targeted, periodized training program and frequent, individualized sessions may mitigate physiological differences previously attributed primarily to biological maturation [31]. Although it has been established that training at VO_2_max intensity, applied in longer and continuous bouts, is more effective for developing endurance—and that reductions in pitch size, by increasing internal load, can elevate players’ VO_2_max values by up to ~10%—from the perspective of training periodization it remains essential to emphasize the structural requirements of football [32]. In particular, the frequent incorporation of small-sided game (SSG) formats is justified, not only for their tactical and technical benefits but also because of their mechanical demands (accelerations, decelerations), which effectively increase metabolic processes and internal load [33,34,35].

From a practical perspective, the position-specific differences observed between the youngest and young adult groups (defenders vs. attackers) highlight the need for fine-tuned, role-specific adjustments to both training prescription and recovery. The faster lactate response typically observed in adolescents may allow for the integration of more intensive stimuli, provided that recovery intervals are appropriately timed and closely monitored [36].

## 5. Conclusions

Following a maximal incremental treadmill test, BLC_max_ was highest in the 15-year-old group and lowest in young adults; however, no significant differences were found between the groups. In position-specific analyses, there was a real difference between defenders and attackers in the youngest and young adult age groups. Overall, our results suggest that, with similar relative muscle mass and aerobic capacity, age-related differences are primarily attributable to transport and elimination processes rather than lactate production capacity. The faster lactate kinetics observed in adolescents suggest that, compared to traditional adult protocols, recovery intervals may be reduced by 15–20% without compromising training quality. In practice, this supports the individualization of age- and position-specific training programs and the precise timing of recovery [36], especially in the development of young people. Therefore, the amount of training and recovery should be selected with greater precision, especially in these contexts. These findings support position-specific training adjustments, particularly for defenders and forwards in younger age categories.

**Limitations:** During the BLC assessment, only two samples were collected within the same time interval, which precluded consideration of serum delay time; this limits the conclusions that can be drawn regarding the decline phase of BLC. The relatively narrow age range reflected the constraints of the educational system; the inclusion of younger cohorts could have biased the results as their training methods and load patterns differ substantially from those of older players. Future research should incorporate longitudinal follow-up from puberty into young adulthood and specifically investigate gender differences, given the distinct hormonal profiles and muscle fiber characteristics of female athletes. The use of modern monitoring technologies, such as wearable devices and real-time lactate tracking, may provide deeper insights into the metabolic demands of match play [37]. Overall, although adolescents do not possess a “secret weapon” in lactate metabolism [11]; their kinetic characteristics result in distinct training responses. Consequently, training volume and recovery should be tailored with greater precision, particularly during the developmental years and in the context of talent identification.

## Figures and Tables

**Table 1:** Study sample by age groups and positions (*n* = 147).

**Age Groups**	**n**	**%**
U15	49	33.3
U16	45	30.6
U17	30	20.4
U18/U19	23	15.7
**Positions**	**n**	**%**
defenders	49	33.3
midfielders	40	27.2
forwards	41	27.9
goalkeepers	17	11.6

**Table 2:** Anthropometric and body composition characteristics by the age groups.

	U15		U16		U17		U18/U19		Post Hoc
	M	SD	M	SD	M	SD	M	SD	
BH (cm)	175.16	9.86	177.28	7.30	179.29	8.32	182.58	5.46	U15 = U16 < U18/U19
BW (kg)	61.86	10.87	64.13	8.46	68.49	8.84	72.03	7.08	U15 = U16 < U17 = U18/U19
M%	44.02	3.72	43.55	1.85	43.96	1.75	43.39	1.96	ns
F%	8.10	3.2	8.65	3.28	9.11	3.48	10.86	3.64	U15 = U16 < U18/U19

Notes. BH = body height (cm), BW = body weight (kg), M% = relative muscle mass, F% = relative fat mass, ns = non-significant differences.

**Table 3:** Results of exercise tests in the laboratory by the age groups.

	U15		U16		U17		U18/U19		F	*p* *	ω^2^	Post Hoc
	M	SD	M	SD	M	SD	M	SD				
VO_2max_ (mL/min)	3264.53	583.00	3490.76	557.87	3894.90	472.25	4136.87	504.98	17.833	<0.001	0.25	U18/U19 = U17 > U15 = U16
HRAT (beat/min)	180.86	12.46	181.18	10.63	170.83	13.51	174.78	9.74	5.722	0.001	0.10	U15 = U16 > U17, U18/U19 *
rVO_2max_ (mL/kg/min)	57.05	6.64	56.41	6.80	58.02	4.39	58.54	5.79	0.830	0.482	0.00	ns
rBLC (mmol/L)	1.13	0.32	1.14	0.30	1.46	0.67	1.43	0.42	4.975	0.004	0.09	U18/U19 > U15 = U16, U17 *
IBLC (mmol/L)	17.92	2.85	16.30	3.36	15.95	3.26	13.91	2.96	10.007	<0.001	0.14	U15 = U16 > U18/U19, U17 *
ΔIBLC (mmol/L)	16.78	2.86	15.16	3.28	14.49	3.62	12.48	3.02	11.348	<0.001	0.16	U15 = U16 > U17 = U18/U19
RHR (beat/min)	76.39	7.31	74.58	8.78	78.20	6.62	71.74	10.27	2.805	0.046	0.04	ns
HR_max_ (beat/min)	196.42	6.79	196.78	7.65	195.63	7.39	191.09	6.05	**4.** **654**	**0.** **005**	**0.05**	U15 = U16 > U18/U19, U17 *
Time (s)	666.0	51.2	704.2	67.7	714.0	78.5	646.0	40.8	0.902	0.46	0.02	ns
Speed_max_ (km/h)	16.8	0.7	17.1	0.9	18.1	1.7	16.6	1.0	1.103	0.374	0.17	ns
rPO (W/kg)	4.9	0.3	4.9	0.1	4.7	0.1	4.9	0.1	1.245	0.331	0.08	ns

Notes. VO_2max_ = absolute aerobic capacity (mL/min); HRAT = heart rate at anaerobic threshold (beat/min); rVO_2max_ = relative aerobic capacity (mL/kg/min); rBLC = resting blood lactate (mmol/L); iBLC = immediate blood lactate (mmol/L); ΔIBLC = delta lactate (mmol/L); RHR = resting heart rate (beat/min); HR_max_ = maximal heart rate (beat/min); Time = time spent on the treadmill (s); Speed_max_ = maximum speed achieved during the exercise (km/h); rPO = relative power output (W/kg); ns = non-significant differences; *: intermediate value; bold: significant differences based on adjusted *p*-value (Bonferroni correction).

## Data Availability

The data presented in this study are available on request from the corresponding author without undue reservation.

## Abbreviations

The following abbreviations are used in this manuscript:

VO_2_max	Absolute aerobic capacity (mL/min)
rVO_2_max	Relative aerobic capacity (mL/kg/min)
HRAT	Heart rate at anaerobic threshold (beat/min)
RHR	Resting heart rate (beat/min)
HR_max_	Maximal heart rate (beat/min)
rBLC	Resting blood lactate (mmol/L)
iBLC	Immediate blood lactate (mmol/L)
ΔiBLC	Delta lactate (mmol/L)
Time	Time spent on the treadmill (s)
Speedmax	Maximum speed achieved during the exercise (km/h)
rPO	Relative level of performance (W/kg)
BH	Body height (cm)
BW	Body weight (kg)
M%	Relative muscle mass
F%	Relative fat mass

## References

[1] Hebestreit H., Mimura K., Bar-Or O. (1993). Recovery of muscle power after high-intensity short-term exercise: Comparing boys and men. J. Appl. Physiol..

[2] Boisseau N., Delamarche P. (2000). Metabolic and hormonal responses to exercise in children and adolescents. Sports Med..

[3] Taylor D.J., Kemp G.J., Thompson C.H., Radda G.K. (1997). Ageing: Effects on oxidative function of skeletal muscle in vivo. Mol. Cell. Biochem..

[4] Tonson A., Ratel S., Le Fur Y., Cozzone P.J., Bendahan D. (2008). Effect of maturation on the relationship between muscle size and force production. Med. Sci. Sports Exerc..

[5] Ratel S., Duché P., Williams C.A. (2002). Muscle fatigue during high-intensity exercise in children. Sports Med..

[6] Dotan R., Mitchell C., Cohen R., Klentrou P., Gabriel D., Falk B. (2003). Child–adult differences in muscle activation—A review. Pediatr. Exerc. Sci..

[7] Zafeiridis A., Chatziioannou A.C., Paraschos I. (2005). Energy system contributions and physiological responses during high-intensity intermittent exercise in prepubertal and pubertal boys. J. Strength. Cond. Res..

[8] Ratel S., Blazevich A.J. (2017). Are prepubertal children metabolically comparable to well-trained adult endurance athletes?. Sports Med..

[9] Dotan R., Falk B. (2011). Children’s repeated sprint ability: Non-metabolic influences on performance. Int. J. Sports Physiol. Perform..

[10] van Praagh E., Doré E. (2002). Short-term muscle power during growth and maturation. Sports Med..

[11] Falk B., Dotan R. (2006). Child–adult differences in the recovery from high-intensity exercise. Exerc. Sport Sci. Rev..

[12] Armstrong N., McManus A.M. (2011). Physiology of elite young male athletes. Med. Sport Sci..

[13] Malina R.M., Bouchard C., Bar-Or O. (2004). Growth, Maturation, and Physical Activity.

[14] Mancera-Soto E.M., Schmidt W.F., Schmidt W., Friedmann-Bette B., Wachsmuth N.B. (2022). Hemoglobin Mass, Blood Volume, and VO_2_max of Trained and Untrained Children and Adolescents Living at Different Altitudes. Int. J. Environ. Res. Public Health.

[15] Beneke R. (2005). Modeling the blood lactate kinetics at maximal short-term exercise conditions in children, adolescents, and adults. J. Appl. Physiol..

[16] Engel F.A., Sperlich B., Stockinger C., Hahn L., Mester J. (2015). The kinetics of blood lactate in boys during and following a single and repeated all-out sprints of cycling are different than in men. Appl. Physiol. Nutr. Metab..

[17] Dotan R. (2015). Discussion: The kinetics of blood lactate in boys during and following a single and repeated all-out sprints of cycling are different than in men. Appl. Physiol. Nutr. Metab..

[18] Bangsbo J. (2014). Physiological demands of football. Sports Sci. Exch..

[19] Altmann S., Ringhof S., Neumann R., Woll A., Rumpf M.C. (2020). Endurance capacities in professional soccer players: Are there differences between positions?. Sports.

[20] Slimani M., Nikolaidis P.T., Dellal A., Chaabene H. (2019). Effects of training programs on physical and physiological aspects in soccer players: A systematic review. Sports.

[21] Buchheit M., Mendez-Villanueva A. (2014). Physical capacity–match physical performance relationships in soccer: Simply, more complex. Eur. J. Appl. Physiol..

[22] Buchheit M., Mendez-Villanueva A. (2014). Changes in repeated-sprint performance in relation to change in locomotor profile in highly trained young soccer players. J. Sports Sci..

[23] Marfell-Jones M., Stewart A., de Ridder J. (2012). International Standards for Anthropometric Assessment.

[24] Sun G., French C.R., Martin G.R., Younghusband B., Green R.C., Xie Y.G., Mathews M., Barron J.R., Fitzpatrick D.G., Gulliver W. (2005). Comparison of multifrequency bioelectrical impedance analysis with dual-energy X-ray absorptiometry for assessment of body composition in a population-based study. Am. J. Clin. Nutr..

[25] Padulo J., Powell D., Milia R., Ardigò L.P. (2013). A Paradigm of Uphill Running. PLoS ONE.

[26] Beaver W.L., Wasserman K., Whipp B.J. (1986). A new method for detecting anaerobic threshold by gas exchange. J. Appl. Physiol..

[27] Tanaka H., Monahan K.D., Seals D.R. (2001). Age-predicted maximal heart rate revisited. J. Am. Coll. Cardiol..

[28] Brink-Elfegoun T., Kaijser L., Gustafsson T., Ekblom B. (2007). Maximal oxygen uptake is not limited by a central nervous system governor. J. Appl. Physiol..

[29] Åstrand P.O., Rodahl K. (1986). Textbook of Work Physiology: Physiological Bases of Exercise.

[30] Ratel S., Lazaar N., Doré E., Baquet G., Williams C.A., Berthoin S., Duché P. (2008). High-intensity intermittent activities at school: Age-related differences in repeated sprint performance in 11- to 15-year-old boys. Eur. J. Appl. Physiol..

[31] Slimani M., Nikolaidis P.T. (2019). Anthropometric and physiological characteristics of male soccer players according to their competitive level, playing position and age group: A systematic review. J. Sports Med. Phys. Fitness.

[32] Köklü Y., Asçi A., Koçak F.Ü., Alemdaroglu U., Dündar U. (2011). Comparison of the physiological responses to different small-sided games in elite young soccer players. J. Strength. Cond. Res..

[33] Kelly D.M., Drust B. (2009). The effect of pitch dimensions on heart rate responses and technical demands of small-sided soccer games in elite players. J. Sci. Med. Sport.

[34] Hill-Haas S.V., Dawson B.T., Coutts A.J., Rowsell G.J. (2009). Physiological responses and time characteristics of various small-sided soccer games in youth players. J. Sports Sci..

[35] Rudarlı G., Tutar M., Kayitken B. (2024). Effects of various endurance training models on the physical condition of football players during the national break of the season. Acta Kinesiol..

[36] Thom G., Kavaliauskas M., Babraj J. (2020). Changes in lactate kinetics underpin soccer performance adaptations to cycling-based sprint interval training. Eur. J. Sport Sci..

[37] Granacher U., Lesinski M., Büsch D., Muehlbauer T., Prieske O., Puta C., Behm D.G. (2016). Effects of resistance training in youth athletes on muscular fitness and athletic performance: A conceptual model for long-term athlete development. Front. Physiol..

